# Availability and threshold of the vasoactive-inotropic score for predicting early extubation in adults after rheumatic heart valve surgery: a single-center retrospective cohort study

**DOI:** 10.1186/s12871-024-02489-7

**Published:** 2024-03-18

**Authors:** Yang Zhao, Hanlei Zhao, Jiao Huang, Bo Mei, Jun Xiang, Yizheng Wang, Jingyan Lin, San Huang

**Affiliations:** 1 Department of anesthesiology, Affiliated Hospital of North Sichuan Medical College, Nanchong, China; 2 Department of anesthesiology, First Affiliated Hospital of GuangXi Medical University, Nanning, China; 3 Department of anesthesiology, Langzhong Hospital of Traditional Chinese Medicine, Langzhong, China; 4 Department of cardiovascular surgery, Dazhou Central Hospital, Dazhou, China; 5 Department of cardiovascular surgery, Affiliated Hospital of North Sichuan Medical College, Nanchong, China

**Keywords:** Vasoactive-inotropic score, Early extubation, Rheumatic, Valve, Cardiopulmonary bypass

## Abstract

**Background:**

Early extubation (EEx) is defined as the removal of the endotracheal tube within 8 h postoperatively. The present study involved determining the availability and threshold of the vasoactive-inotropic score (VIS) for predicting EEx in adults after elective rheumatic heart valve surgery.

**Methods:**

The present study was designed as a single-center retrospective cohort study which was conducted with adults who underwent elective rheumatic heart valve surgery with CPB. The highest VIS in the immediate postoperative period was used in the present study. The primary outcome, the availability of VIS for EEx prediction and the optimal threshold value were determined using ROC curve analysis. The gray zone analysis of the VIS was performed by setting the false negative or positive rate *R* = 0.05, and the perioperative risk factors for prolonged EEx were identified by multivariate logistic analysis. The postoperative complications and outcomes were compared between different VIS groups.

**Results:**

Among the 409 patients initially screened, 379 patients were ultimately included in the study. The incidence of EEx was determined to be 112/379 (29.6%). The VIS had a good predictive value for EEx (AUC = 0.864, 95% CI: [0.828, 0.900], *P* < 0.001). The optimal VIS threshold for EEx prediction was 16.5, with a sensitivity of 71.54% (65.85–76.61%) and a specificity of 88.39% (81.15–93.09%). The upper and lower limits of the gray zone for the VIS were determined as (12, 17.2). The multivariate logistic analysis identified age (OR, 1.060; 95% CI: 1.017–1.106; *P* = 0.006), EF% (OR, 0.798; 95% CI: 0.742–0.859; *P* < 0.001), GFR (OR, 0.933; 95% CI: 0.906–0.961; *P* < 0.001), multiple valves surgery (OR, 4.587; 95% CI: 1.398–15.056; *P* = 0.012), and VIS > 16.5 (OR, 12.331; 95% CI: 5.015–30.318; *P* < 0.001) as the independent risk factors for the prolongation of EEx. The VIS ≤ 16.5 group presented a greater success rate for EEx, a shorter invasive ventilation support duration, and a lower incidence of complications than did the VIS > 16.5 group, while the incidence of reintubation was similar between the two groups.

**Conclusion:**

In adults, after elective rheumatic heart valve surgery, the highest VIS in the immediate postoperative period was a good predictive value for EEx, with a threshold of 16.5.

**Supplementary Information:**

The online version contains supplementary material available at 10.1186/s12871-024-02489-7.

## Background

Rheumatic heart disease (RHD) affects more than 30 million people worldwide, causing approximately 300,000 deaths and 10 million disabilities each year [1]. In low-income and developing countries such as China, RHD is a major cause of morbidity and mortality [2, 3]. The development of strategies to improve the prognosis of patients with RHD is highly important.

Early extubation (EEx) was defined as the removal of the endotracheal tube within 8 h of admission to the intensive care unit (ICU) after the completion of surgery [4]. Previous studies suggest the close association of the EEx with the lower intensive care unit (ICU) and length of hospital stay (LOS), decreased hospitalization expenses, and reduced mortality in patients undergoing cardiac pulmonary bypass (CPB) surgery [5–7]. EEx reportedly does not increase the risk of reintubation after aortic valve replacement surgery [8]. These findings have led to the widespread use of EEx protocols among surgeons, anesthesiologists, and ICUs for patients undergoing coronary artery bypass grafting and valve surgery [9, 10].

Gaies et al. proposed the use of the vasoactive-inotropic score (VIS), which is a weighted sum of all administered inotropes and vasoconstrictors, including dopamine, dobutamine, epinephrine, norepinephrine, milrinone, vasopressin, and levosimendan, for accurate measurement of cardiovascular dysfunction and prediction of outcomes in infants after cardiopulmonary bypass [11]. Recent studies have reported that the VIS is correlated with mortality in patients with sepsis and cardiac surgery [12, 13]. Moreover, the highest VIS in the immediate postoperative period was revealed a predictive factor for poor outcomes [14]. However, to the best of our knowledge, no study thus far has used the highest VIS in the immediate postoperative period for predicting EEx in the ICU for adult patients with elective rheumatic heart valve bypass surgery.

In this context, the present retrospective cohort study aimed to evaluate whether the highest VIS in the immediate postoperative period is an independent predictor of EEx and to determine the diagnostic threshold value of the VIS among adult patients who underwent elective rheumatic heart valve bypass surgery.

## Methods

### Study procedure and patients

**Fig. 1:**
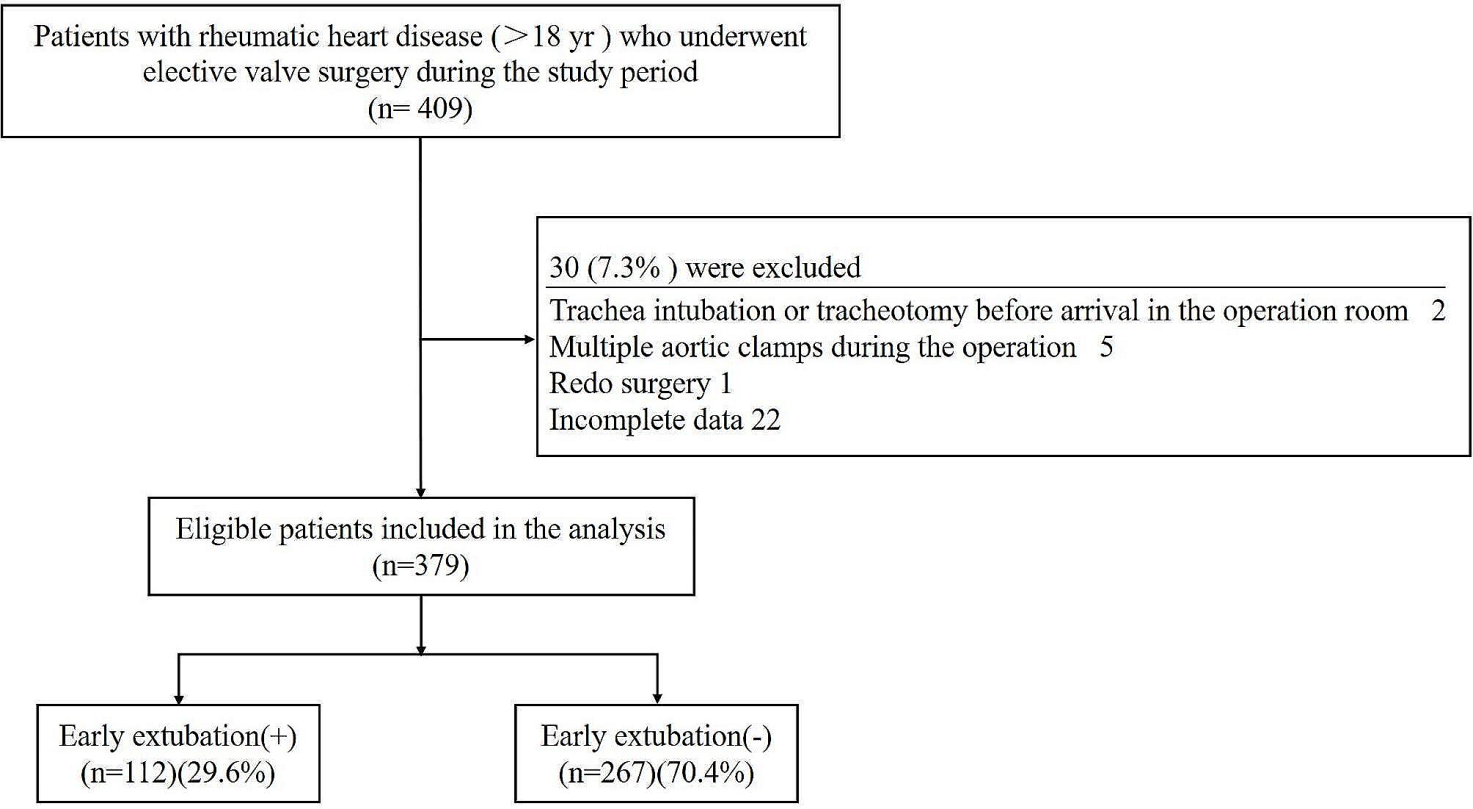
Flow chart of the study procedure

The present study was designed as a single-center, retrospective, cohort study conducted between January 2017 and December 2021. The study procedure was approved by the Medical Ethics Committee of the Affiliated Hospital of North Sichuan Medical College. The study is registered with the Chinese Registry of Clinical Trials (http://www.chictr.org.cn) (ChiCTR2300071659; 22/05/2023) and was conducted in accordance with the Declaration of Helsinki (2013). The requirement for obtaining written informed consent from each participant was exempted from the Ethics Committee due to the retrospective nature of the study, which ensured no exposure risks to the patients due to any additional intervention. The inclusion criteria for participation in the study were age > 18 years and a history of elective rheumatic heart valve surgery with CPB. The exclusion criteria included unplanned tracheal intubation prior to surgery, preoperative EF < 30%, persistent heart failure prior to surgery, preoperative plasma creatinine > 2.0 mg/dL, cardiac arrest prior to CPB, multiple aortic clampings, additional coronary artery bypass grafting (CABG) procedures, and redo surgery until discharge from the hospital. During this period, 409 consecutive adult patients who underwent elective rheumatic heart valve surgery were screened in the present study. Among these, 30 (7.3%) patients were excluded for the following reasons: tracheal intubation or tracheotomy prior to arrival in the operating room (*n* = 2), multiple aortic clamps during surgery (*n* = 5), redo surgery (*n* = 1), or incomplete data (*n* = 22). Finally, 379 patients were enrolled in the study for analysis. The flow chart of the study procedure is depicted in Fig. 1. The eligible patients were divided into two groups, namely, the success group for EEx (+) and the prolongation group for EEx (–) group, based on whether the duration to extubation exceeded 8 h or not. The postoperative complications and outcomes were compared between the different VIS groups by dividing the patients into two groups based on the optimal VIS threshold determined via ROC curve analysis.

### Data collection

Patient demographics, intraoperative data, and postoperative variables were extracted from the medical records available at our hospital (details are shown in Table 1).

**Table 1:** Patient demographics and preoperative data

	Variables	All patients (*n* = 379)	Early extubation (+) (*n* = 112)	Early extubation (-) (*n* = 267)	*P* value
	Time to extubation(h)	14.6 ± 18.0	7.1 ± 0.6	17.7 ± 20.6	< 0.001
Patient characteristics	Male	160 (42.2%)	50 (44.6%)	110 (41.2%)	0.570
	Age (y)	56.2 ± 10.9	50.4 ± 11.1	58.6 ± 9.8	< 0.001
	BMI	22.8 ± 3.0	23.0 ± 3.0	22.7 ± 3.0	0.444
	Hypertension	54 (14.2%)	16 (14.3%)	38 (14.2%)	1.000
	COPD	35 (9.3%)	7 (6.3%)	28 (10.5%)	0.245
	History of smoking	99 (26.1%)	31 (27.7%)	68 (25.5%)	0.701
	Diabetes	24 (6.3%)	5 (4.5%)	19 (7.1%)	0.369
Preoperative factors	ASA classification (II/ III/ IV)	16/ 351/ 12 (4.2%/92.6%/3.2%)	9/103/0 (8.0%/ 92.0%/0)	7/248/12 (2.6%/92.9%/4.5%)	0.004
	EF(%)	53.5 ± 7.7	60.1 ± 5.6	50.7 ± 6.7	< 0.001
	GFR (ml/min*1.73m^2^)	71.2 ± 16.4	82.1 ± 19.7	66.7 ± 12.3	< 0.001
	Hb (g/L)	126.5 ± 18.0	128.2 ± 16.4	125.8 ± 18.5	0.223
	PaO2 (mmHg)	81.1 ± 8.5	81.8 ± 7.4	80.7 ± 8.9	0.209
	PaCO2 (mmHg)	42.4 ± 5.6	43.0 ± 5.7	42.1 ± 5.5	0.190
	Fasting blood glucose (mmol/L)	5.4 ± 1.4	5.3 ± 1.0	5.5 ± 1.6	0.206
	PLT (10^9^/L)	178.0 ± 62.6	176.8 ± 62.3	178.4 ± 62.9	0.818
Intraoperative factors	Mitral valve stenosis classification (none/mild/moderate/severe)	81/73/95/130 (21.4%/19.3%/25.1%/34.3%)	38/16/24/34 (33.9%/14.3%/21.4%/30.4%)	43/57/71/96 (16.1%/21.3%/26.6%/36.0%)	0.002
	Mitral valve regurgitation classification (none/mild/moderate/severe)	46/94/110/129 (12.1%/24.8%/29.0%/34.0%)	19/34/28/31 (17.0%/30.4%/25.0%/27.7%)	27/60/82/98 (10.1%/22.5%/30.7%/36.7%)	0.050
	Aortic valve stenosis classification (none/mild/moderate/severe)	207/46/40/86 (54.6%/12.1%/10.6%/22.7%)	55/25/7/25 (49.1%/22.3%/6.3%/22.3%)	152/21/33/61 (56.9%/7.9%/12.4%/22.8%)	< 0.001
	Aortic valve regurgitation classification (none/mild/moderate/severe)	151/144/54/30 (39.8%/38.0%/14.2%/7.9%)	55/33/18/6 (49.1%/29.5%/16.1%/5.4%)	96/111/36/24 (36.0%/41.6%/13.5%/9.0%)	0.043
	Isolated mitral valve repair	29 (7.7%)	11 (9.8%)	18 (6.7%)	0.303
	Isolated mitral valve replacement	298 (78.6%)	72 (64.3%)	226 (84.6%)	< 0.001
	Isolated aortic valve replacement	157 (41.4%)	39 (34.8%)	118 (44.2%)	0.091
	Multiple valve surgery	105 (27.7%)	10 (8.9%)	95 (35.6%)	< 0.001
	Tricuspid valve repair	285 (75.2%)	73 (65.2%)	212 (79.4%)	0.004
	Maze	165 (43.5%)	28 (25.0%)	137 (51.3%)	< 0.001
	Pacemaker implantation	91 (24.0%)	15 (13.4%)	76 (28.5%)	0.002
	Ultrafiltration (ml)	775.4 ± 968.3	624.5 ± 831.6	838.7 ± 1015.0	0.049
	ACC time (min)	100.7 ± 33.0	87.1 ± 26.1	106.5 ± 34.0	< 0.001
	CPB time (min)	151.4 ± 41.3	132.5 ± 32.3	159.3 ± 42.1	< 0.001
	Lowest Hb(g/L)	71.2 ± 9.9	71.6 ± 7.9	71.0 ± 10.6	0.500
	Highest Lac (mmol/L)	3.4 ± 1.6	2.8 ± 1.0	3.7 ± 1.8	< 0.001
	Highest blood glucose (mmol/L)	9.8 ± 3.0	9.5 ± 2.7	9.9 ± 3.2	0.219
	Hemorrhage (ml)	457.0 ± 94.3	419.2 ± 59.4	472.9 ± 101.5	< 0.001
	Crystolloid infusion (ml)	932.3 ± 323.8	900.0 ± 298.6	945.9 ± 333.4	0.209
	Colloid infusion (ml)	438.8 ± 140.0	441.5 ± 128.0	437.6 ± 144.8	0.806
	VIS	18.4 ± 7.5	13.9 ± 2.2	20.3 ± 8.0	< 0.001

After the patients were weaned from CPB, vasoactive-inotropic agents were titrated to maintain the desired hemodynamic stability. After surgery, the VIS was measured every 5 min or more frequently, as indicated by the change in dose or the addition of drugs from the medical records. The highest VIS in the immediate postoperative period was selected for analysis in the present study. VIS was calculated as described in a previous report [15], using the following formula:

VIS = dopamine (µg/kg/min) + dobutamine (µg/kg/min) + 100 × epinephrine (µg/kg/min) + 100 × norepineprine (µg/kg/min) + 10 × milrinone (µg/kg/min) + 10,000 × vasopressin (units/kg/min) + 50 × levosimendan (µg/kg/min).

### Extubation plan

After rheumatic heart valve surgery, patients were sent to the cardiac ICU, where they were placed on continuous invasive ventilation. Patients were managed according to the enhanced recovery protocol, which included lower-dose opioids, limited perioperative fluids, and accelerated physical rehabilitation on the morning after the operation [16]. The patient was considered ready for extubation immediately upon fulfilling the extubation criteria. The following extubation criteria were used, according to previous studies [17, 18]:

(1) Hemodynamic stability with reasonable urination and warm peripheral extremities;

(2) PaO_2_ ≥ 80 mmHg and PaCO_2_ ≤ 50 mmHg with adequate spontaneous respiration, FiO_2_ ≤ 0.4, and end-expiratory pressure ≤ 5 cm H_2_O;

(3) Awake and able to respond to commands, with no new neurological symptoms observed;

(4) No active bleeding, with stable hemoglobin levels and no requirement for volume replacement;

(5) Patients had no reasonable fear of reintubation.

### Outcome measures

The primary outcome of the present study was the availability and threshold of the VIS for predicting EEx. The gray zone analysis of the VIS was performed, and the perioperative risk factors for prolonged EEx were identified by multivariate logistic analysis. Postoperative complications and outcomes, including pneumonia, cardiac dysfunction, IABP, cardiopulmonary resuscitation (CPR), acute kidney injury (AKI), death at the hospital, reintubation, ICU stay, and length of stay (LOS) after surgery, were compared between the different VIS groups.

### Statistics

A 44.3% incidence of EEx was assumed based on a previous study [19]. In addition, a 5% acceptable margin of error and a 95% confidence interval (CI) were used. The appropriate sample size for the study was calculated to be [1.96^2^*0.443*(1-0.443)]/0.05^2^= 379. Categorical variables are expressed as the frequency (n) and the corresponding percentage (%) and were analyzed using the two-sided Fisher exact test or chi-squared test, as appropriate. Continuous variables are expressed as the mean ± standard deviation (SD) and were evaluated using the two-sample Student’s t-test. The availability of the VIS to predict EEx and the optimal threshold value were determined using receiver operating characteristic (ROC) curve analysis.

Due to the wide range of VIS, it is quite difficult for some patients to predict perfectly EEx (+) or EEx (–). To address this problem, it is necessary to propose a three-zone partition (negative, positive, and the gray zone between them), which was constructed from the cutoff value of the VIS [20]. In the gray zone, VIS cannot be precisely predicted as EEx (+) or EEx (–). The upper or lower limit of the gray zone was determined by setting the false negative or positive rate *R* = 0.05.

The risk factors with *P* < 0.05 revealed in the univariate logistic analysis were subjected to multivariate logistic analysis to identify the risk factors for prolonged EEx. Statistical analysis was conducted using SPSS 25.0 software (Statistical Program for Social Sciences, SPSS, Inc., Chicago, Illinois, USA), considering *P* < 0.05 as the threshold of statistical significance.

## Results

### Patient characteristics

As presented in Table 1, patients in the EEx (+) group were younger than those in the EEx (–) group (50.4 ± 11.1 vs. 58.6 ± 9.8 years, *P* < 0.001). With respect to preoperative factors, EEx (+) patients were more likely to have a lower ASA classification, greater EF%, and higher GFR compared to the EEx (–) patients.

Considering the intraoperative factors, in the EEx (+) group, compared to those in the EEx (–) group patients, the proportion of moderate-severe stenosis or regurgitation of the mitral or aortic valve were lower, and those were less likely to undergo isolated mitral valve replacement and multiple valve surgery. No differences were observed between the two groups with regard to the proportion of patients who underwent isolated mitral valve repair or isolated aortic replacement surgery. Among the additional procedures, tricuspid valve repair, MAZE, and pacemaker implantation were performed less frequently in the EEx (+) group compared to the EEx (–) group. Moreover, other factors, such as ultrafiltration, ACC duration, CPB duration, highest Lac level, and hemorrhage, were lower in the EEx (+) group. The VIS was much lower in the EEx (+) group compared to the EEx (–) group (13.9 ± 2.2 vs. 20.3 ± 8.0, *P* < 0.001).

### Determining the optimal VIS threshold for predicting EEx

**Fig. 2:**
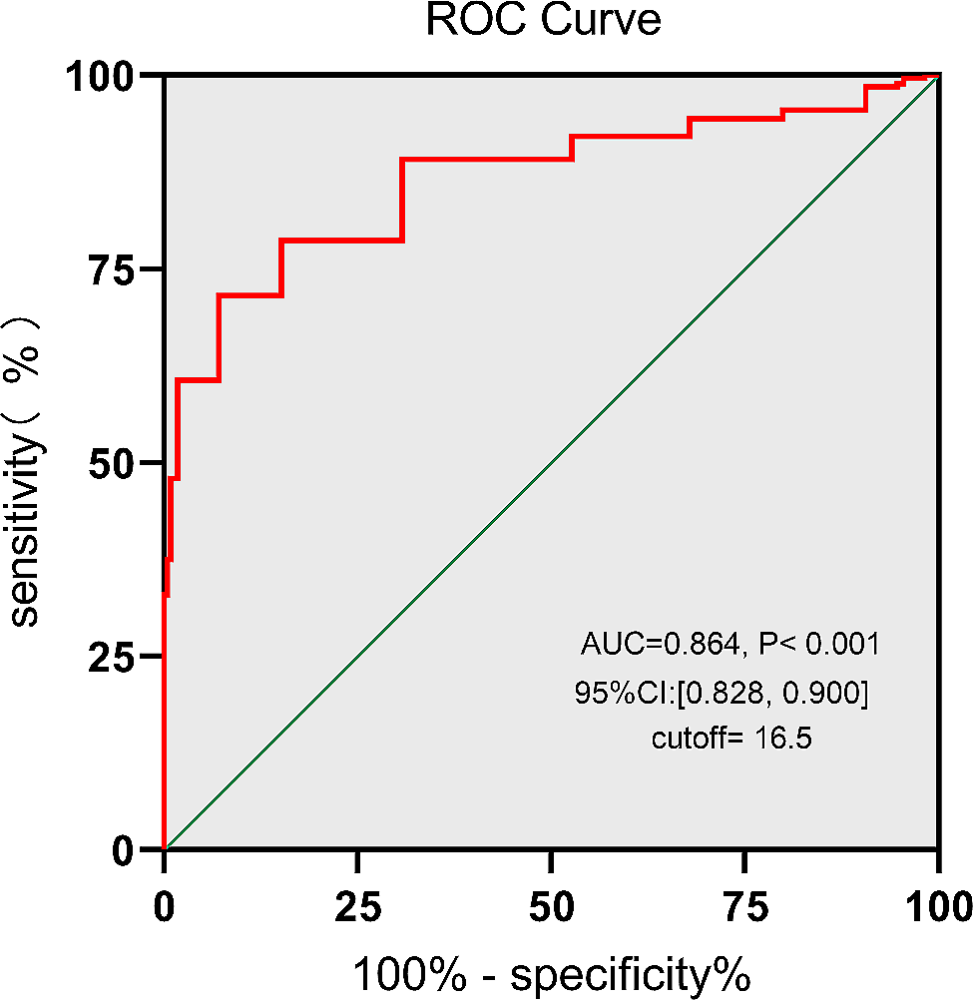
Receiver operating characteristic (ROC) curve analysis of the ability of the VIS to predict EEx. Note: The AUC for the prediction of EEx was 0.864, 95% CI: [0.828, 0.900], *P* < 0.001. The optimal VIS threshold for EEx prediction was 16.5, with a sensitivity of 71.54% (65.85–76.61%) and a specificity of 88.39% (81.15–93.09%)

ROC curve analysis of the VIS for EEx prediction was performed for all patients. As depicted in Fig. 2, the VIS had a good predictive value for EEx (AUC = 0.864, 95% CI: [0.828, 0.900], *P* < 0.001). The optimal VIS threshold for EEx prediction was determined to be 16.5, with a sensitivity of 71.54% (65.85–76.61%) and a specificity of 88.39% (81.15–93.09%). To explore the effect of different valvular pathologies on the prediction value of EEx, we further performed subgroup analyses according to isolated mitral valve surgery, isolated aortic valve surgery, and multiple valve surgery. Similarly, the VIS had good predictive values for EEx among all subgroups (isolated mitral valve surgery: AUC = 0.8808, 95% CI: [0.8366, 0.9250], *P* < 0.001, cutoff = 16.5; isolated aortic valve surgery: AUC = 0.7631, 95% CI: [0.6184, 0.9078], *P* = 0.0012, cutoff = 17.5; multiple valve surgery: AUC = 0.8689, 95%CI: [0.7878, 0.9501], *P* = 0.001, cutoff = 15.5) (Shown in supplementary material1-3). Next, the patients were classified into two groups based on the optimal VIS threshold: the VIS ≤ 16.5 group (*n* = 175) and the VIS >16.5 group (*n* = 204).

### Analysis of the upper and lower limits of the gray zone for VIS

**Fig. 3:**
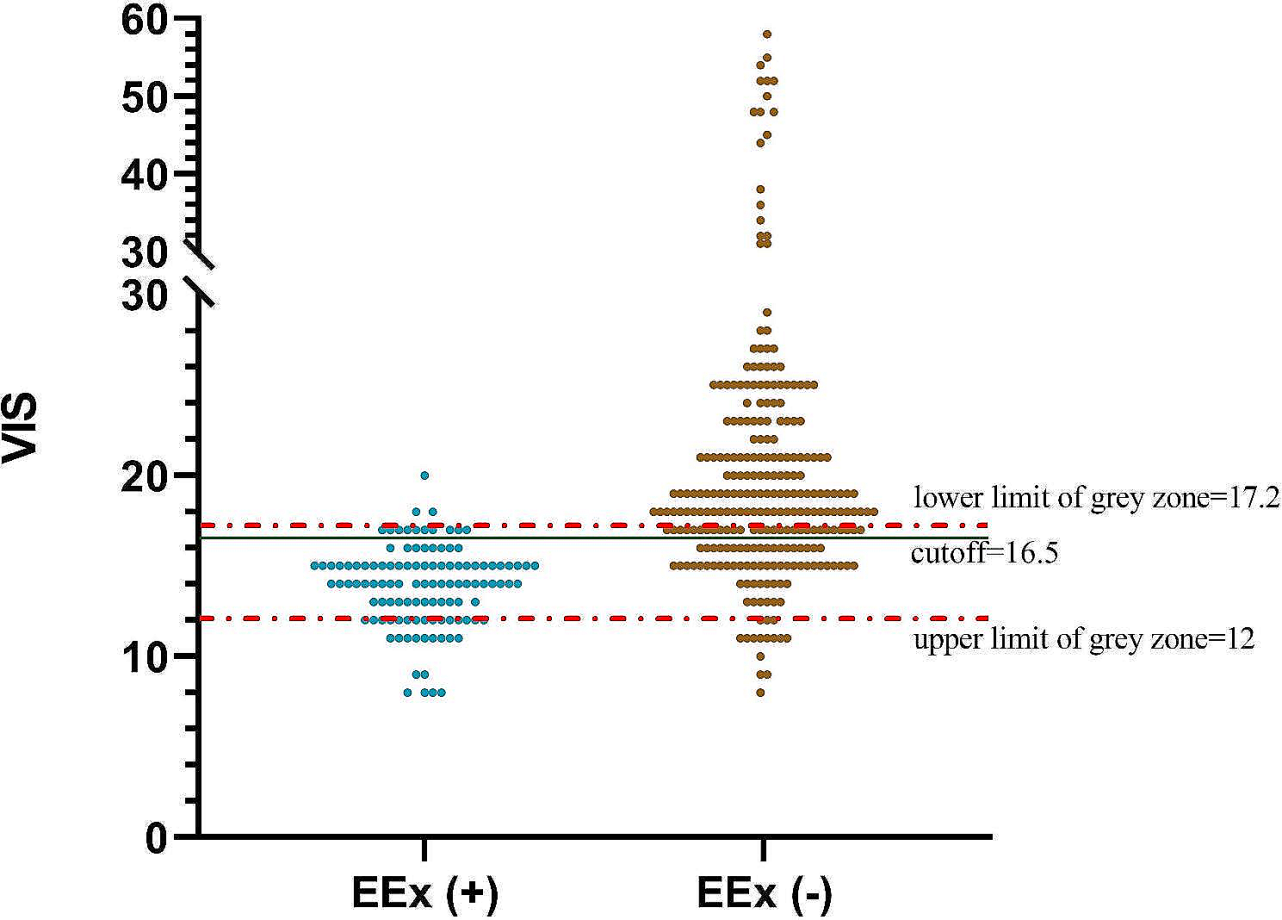
The upper and lower limits of the gray zone for the VIS

The sensitivity, specificity, false positive rate, and false negative rate of the VIS values near the upper and lower limits of the gray zone are shown in Table 2. The upper limit of the gray zone is clearly between 11.5 (P1) and 12.5 (P2) because the false negative rate 0.05 (R) is located between 0.0449 (R1) and 0.0562 (R2); correspondingly, the upper limit (P) of the gray zone is calculated to be 12 based on the linear interpolation method: (R1-R2)/(R1-R)=(P1-P2)/(P1-P). Similarly, the lower limit of the gray zone is between 16.5 and 17.5 because the false positive rate 0.05(R) is located between 0.1161 and 0.0268. The lower limit of the gray zone was determined to be 17.2. The gray zone is depicted in Fig. 3, and 187 (49.3%) patients were located in the gray zone.

**Table 2:** The sensitivity, specificity, false positive rate, and false negative rate of VIS values near the upper and lower limits of the gray zone

VIS near the limit of the grey zone	Sensitivity	False positive rate	Specificity	False negative rate
8.5	0.9963	0.96429	0.03571	0.0037
9.5	0.9888	0.94643	0.05357	0.0112
10.5	0.985	0.94643	0.05357	0.015
11.5	0.9551	0.8661	0.1339	0.0449
12.5	0.9438	0.7321	0.2679	0.0562
13.5	0.9213	0.625	0.375	0.0787
14.5	0.8914	0.4286	0.5714	0.1086
15.5	0.7865	0.1875	0.8125	0.2135
16.5	0.7154	0.1161	0.8839	0.2846
17.5	0.6067	0.0268	0.9732	0.3933
18.5	0.4794	0.0089	0.9911	0.5206
19.5	0.3745	0.0089	0.9911	0.6255

### Perioperative risk factors associated with prolonged EEx

To determine the perioperative risk factors associated with prolonged EEx, a univariate logistic analysis was performed, which revealed age, ASA classification, EF%, GFR, mitral valve stenosis classification, mitral valve regurgitation classification, isolated mitral valve replacement, multiple valve surgery, tricuspid valve repair, MAZE, pacemaker implantation, ACC duration > 90 min, CPB duration > 142 min, highest lactate level, hemorrhage > 450 mL, and VIS > 16.5 as the significant variables. These identified significant variables were subsequently subjected to multivariate logistic analysis, which revealed age (OR, 1.060; 95% CI: 1.017–1.106;*P* = 0.006), EF% (OR, 0.798; 95% CI: 0.742–0.859; *P* < 0.001), GFR (OR, 0.933; 95% CI: 0.906–0.961; *P* < 0.001), multiple valves surgery (OR, 4.587; 95% CI: 1.398–15.056; *P* = 0.012), and VIS > 16.5 (OR, 12.331; 95% CI: 5.015–30.318; *P* < 0.001) as the independent risk factors for the prolongation of EEx (Table 3).

**Table 3:** Univariate and multivariate logistic regression analyses conducted to determine the perioperative risk factors for prolonged EEx in patients who underwent elective valve surgery with CPB

		Univariate analysis			Multivariate analysis	
	Variables	OR (95%CI)	*P* value		OR (95%CI)	*P* value
Patient characteristics	Male	0.869 (0.557, 1.356)	0.536		/	/
	Age(y)	1.079 (1.053, 1.105)	< 0.001		1.060 (1.017, 1.106)	0.006
	BMI	0.972 (0.904, 1.045)	0.443		/	/
	Hypertension	0.996 (0.530, 1.871)	0.989		/	/
	COPD	1.757 (0.744, 4.151)	0.199		/	/
	History of smoking	0.893 (0.543, 1.468)	0.655		/	/
	Diabetes	1.640 (0.597, 4.505)	0.338		/	/
Preoperative factors	ASA classification	4.191 (1.680, 10.455)	0.002		1.386 (0.303, 6.342)	0.674
	EF(%)	0.791 (0.751, 0.832)	< 0.001		0.798 (0.742, 0.859)	< 0.001
	GFR (ml/min*1.73m^2^)	0.937 (0.921, 0.954)	< 0.001		0.933 (0.906, 0.961)	< 0.001
	Hb (g/L)	0.992 (0.980, 1.005)	0.223		/	/
	PaCO2 (mmHg)	0.974 (0.936, 1.013)	0.190		/	/
	PaO2 (mmHg)	0.984 (0.958, 1.011)	0.242		/	/
	Glucose (mmol/L)	1.092 (0.926, 1.287)	0.295		/	/
	PLT (10^9^/L)	1.000 (0.997, 1.004)	0.818		/	/
Intraoperative factors	Mitral valve stenosis classification	1.296 (1.068, 1.571)	0.009		0.793 (0.434, 1.450)	0.452
	Mitral valve regurgitation classification	1.335 (1.077, 1.656)	0.008		1.005 (0.631, 1.599)	0.984
	Aortic valve stenosis classification	0.996 (0.835, 1.188)	0.962		/	/
	Aortic valve regurgitation classification	1.245 (0.969, 1.600)	0.087		/	/
	Isolated mitral valve repair	0.664 (0.303, 1.455)	0.306		/	/
	Isolated mitral valve replacement	3.062 (1.839, 5.100)	< 0.001		4.274 (0.771, 23.700)	0.097
	Isolated aortic valve replacement	1.482 (0.938, 2.343)	0.092		/	/
	Multiple valves surgery	5.634 (2.809, 11.301)	< 0.001		4.587 (1.398, 15.056)	0.012
	Tricuspid valve repair	2.059 (1.263, 3.358)	< 0.004		0.604 (0.173, 2.113)	0.430
	Maze	3.162 (1.936, 5.163)	< 0.001		1.490 (0.555, 4.003)	0.429
	Pacemaker implantation	2.573 (1.405, 4.713)	0.002		0.981 (0.332, 2.899)	0.972
	Ultrafiltration > 500 ml	1.304 (0.836, 2.033)	0.242		/	/
	ACC time > 90 min	2.154 (1.368, 3.391)	0.001		1.192 (0.382, 3.719)	0.762
	CPB time > 142 min	2.748 (1.727, 4.373)	< 0.001		1.132 (0.341, 3.753)	0.840
	Lowest hemoglobin(g/L)	0.993 (0.971, 1.016)	0.548		/	/
	Highest Lac (mmol/L)	1.693 (1.363, 2.101)	< 0.001		1.252 (0.881, 1.780)	0.210
	Highest glucose (mmol/L)	1.048 (0.972, 1.131)	0.219		/	/
	Hemorrhage > 450 ml	2.839 (1.690, 4.770)	< 0.001		1.975 (0.758, 5.148)	0.164
	Crystolloid infusion (ml)	1.000 (1.000, 1.001)	0.209		/	/
	Colloid infusion (ml)	1.000 (0.998, 1.001)	0.805		/	/
	VIS > 16.5	19.139 (10.128, 36.164)	< 0.001		12.331 (5.015, 30.318)	< 0.001

### Comparisons of postoperative complications and outcomes between the different VIS groups

The complications and other outcomes after surgery were compared between the different VIS groups. The patients were divided into two groups based on the optimal VIS threshold: the VIS ≤ 16.5 group (*n* = 175) and the VIS > 16.5 group (*n* = 204). As presented in Table 4, the percentage of successful EEx was significantly greater in the VIS ≤ 16.5 group than in the VIS > 16.5 group (88.4% vs. 11.6%, *P* < 0.001). Moreover, the duration of extubation was shorter in the VIS ≤ 16.5 group than in the VIS > 16.5 group (8.8 ± 2.5 h vs. 19.5 ± 23.3 h, *P* < 0.001). With regard to complications, the VIS ≤ 16.5 group presented lower incidences of pneumonia, cardiac dysfunction, IABP, CPR, and AKI; death at the hospital; prolonged ICU stay; and longer LOS after surgery than did the VIS > 16.5 group. On the other hand, the incidence of reintubation was similar between the two groups.

**Table 4:** Comparisons of the postoperative complications and outcomes between patients in different VIS groups

Variables	All patients (*n* = 379)	VIS ≤ 16.5 (*n* = 175)	VIS>16.5 (*n* = 204)	*P* value
Early extubation	112 (29.6%)	99 (88.4%)	13 (11.6%)	< 0.001
Time to extubation (h)	14.6 ± 18.0	8.8 ± 2.5	19.5 ± 23.3	< 0.001
Pneumonia	161 (42.5%)	38 (21.7%)	123 (60.3%)	< 0.001
Cardiac dysfunction	137 (36.1%)	26 (14.9%)	111 (54.4%)	< 0.001
IABP	18 (4.7%)	0 (0%)	18 (8.8%)	< 0.001
CPR	9 (2.4%)	0 (0%)	9 (4.4%)	0.004
AKI	42 (11.1%)	6 (3.4%)	36 (17.7%)	< 0.001
Death in hospital	11 (2.9%)	0 (0%)	11 (5.4%)	0.001
Reintubation	11 (2.9%)	3 (1.7%)	8 (3.9%)	0.235
ICU stay(d)	2.8 ± 0.9	2.4 ± 0.5	3.2 ± 0.9	< 0.001
LOS after surgery(d)	8.5 ± 2.1	7.8 ± 1.4	9.1 ± 2.3	< 0.001

## Discussion

EEx following cardiac surgery was demonstrated to improve outcomes, including pneumonia incidence, ICU stay, LOS, healthcare costs, morbidity, and mortality [16]. Currently, EEx is widely considered the key objective of enhanced recovery in patients who underwent heart valve surgery with CPB [21]. Hiromoto et al. reported that the incidence of EEx was 44.3% for valve surgery patients receiving CPB [19]. In the aforementioned study, the percentages of patients who underwent multiple valve surgeries (3.2% vs. 8.9%), MAZE (9.7% vs. 25.0%), and tricuspid valve repair (22.6% vs. 65.2%) in the EEx (+) group were significantly lower than those in the present study; these differences could have led to the prolongation of ACC and CPB durations, thereby increasing the risk of lung damage by initiating a systemic inflammatory response, hemodilution, intrapulmonary shunt, ischemic damage, ischemia-reperfusion, and atelectasis [22, 23], resulting in the prolongation of mechanical ventilation support. These findings might explain why the incidence of EEx in the present study (29.6%) was lower than that reported by Hiromoto et al [19].

To the best of our knowledge, the present study is the first to explore the availability and threshold of the VIS and its cutoff for the prediction of EEx in adults who underwent elective rheumatic heart valve surgery. The results of the present study indicated that a VIS > 16.5 in the immediate postoperative period was a good indicator of sensitivity and specificity, with an AUC = 0.864, for the independent prediction of EEx in the ICU. This finding is similar to that of Haque et al., who demonstrated that a VIS > 20 was an independent predictive factor for mortality in children with septic shock [24]. However, the gray zone of the VIS was determined to be (12, 17.2), and 49.3% of the patients were located in the gray zone, which indicates that the predicted VIS for EEx was relatively limited. Therefore, VIS located in the gray zone for predicting EEx should be treated conservatively, while perioperative factors, including age, EF%, GFR, and multiple valve surgeries, should be taken into consideration comprehensively to predict EEx.

As vasoplegic syndrome (VS) is frequently observed as a common complication after weaning from CPB, the occurrence of VS varies from 5 to 25% in low-risk cardiac patients; however, in high-risk cardiac patients, vasopressor administration before CPB; prolonged duration of CPB; preoperative low EF; and preoperative administration of beta-blockers or angiotensin-converting enzyme inhibitors, the incidence of VS can increase to a range of 30–50% [25–27]. VS represents a type of distributive shock that is characterized by low systemic vascular resistance and normal-to-high cardiac output, resulting in severe hypotension and increased consumption of vasopressors to maintain adequate systemic perfusion. In addition, in the early postoperative period after being separated from CPB, patients require vasoactive-inotropic agents to support cardiac function, as a high VIS usually represents poor cardiovascular function [14]. Therefore, the VIS directly reflects overall heart dysfunction and low systemic vascular resistance after weaning from CPB in the present study. Due to cardiovascular dysfunction, blood may accumulate in the lungs, facilitating intrapulmonary shunts and impairing gas exchange and pulmonary ventilation function. Moreover, decreased cardiac output may limit perfusion to the coronary, systemic, and microcirculatory systems, resulting in acidosis and inadequate perfusion in multiple organs, including the heart, forming a vicious cycle. Collectively, these factors could explain why a high VIS could be an independent predictor of prolonged EEx. Likewise, patients with a high VIS in the immediate postoperative period should also be allowed to extubate timely in the ICU if their respiratory status is good, the hemodynamics are stable, and meet the extubation criteria [28]. However, caution should be taken when considering that after extubation, persistent high VIS in the ICU may increase the risk of reintubation [29].

The multivariable logistic analysis conducted in the present study indicated that age, EF%, GFR, multiple valve surgery, and VIS > 16.5 were the independent risk factors for EEx failure. It is generally recognized that elderly patients with a decreased ‘physiological reserve’ in multiple organs after valve bypass surgery find it difficult to overcome the perioperative period [30]. In addition, the preoperative GFR was revealed to be an independent predictor of EEx in cardiac surgery patients [31, 32]. The reason for this could be that poor renal function facilitates the release of inflammatory cytokines and impairs endothelial function, leading to an increase in the postoperative burden of vasoactive-inotropic agents and the prolongation of EEx [33, 34]. According to previous studies, age, low EF, and complex procedures were revealed to be correlated with the prolongation of invasive mechanical ventilation support after cardiac bypass surgery [35, 36]. Multiple valve surgery prolonged the duration of CPB, which could have led to increased capillary permeability, resulting in pulmonary edema and prolonged mechanical ventilation, even indirectly affecting the postoperative ICU stay and mortality, which is consistent with the findings of previous reports [37–39].

Ample research has indicated that high VIS was closely associated with poor outcomes, including extended ICU and LOS stay, prolonged mechanical ventilation, requirement for renal replacement therapy, and mortality [11, 14]. Consistently, in the present study, the VIS ≤ 16.5 group presented a greater success rate of EEx; shorter invasive ventilation support duration; and lower incidences of complications, including pneumonia, cardiac dysfunction, IABP, CPR, AKI, death at the hospital, prolonged ICU stay, and extended LOS after surgery, than did the VIS > 16.5 group. However, the incidence of reintubation was similar between the two groups. These results indicated that a VIS ≤ 16.5 was associated with better postoperative outcomes without an increase in the incidence of reintubation.

Like with all related research, the present study has certain limitations. First, the study was retrospective and was therefore susceptible to selection bias and confounding factors. In addition, although overall adherence to optimal vasopressor strategies was ensured to achieve optimal hemodynamics, the selection of vasopressor and inotropic agents, fluid resuscitation strategies, etc., was based on the decisions of different anesthesiologists and ICU doctors, which could impact the VIS and clinical outcomes. Third, the preoperative pulmonary function test (PFT) was not performed in the present study. Preoperative PFT may be important because it could be a confounding factor of EEx; however, its value in predicting the need for mechanical ventilation is controversial [40–42]. Many hospitals, including ours, do not routinely perform PFT before cardiac surgery, especially for patients without a history of pulmonary disease. However, we included the preoperative resting PaO2, PaCO2, and COPD diagnosis to assess preoperative lung function. Finally, the study was conducted in a single center at a tertiary care hospital, which limits the scope of generalizing the obtained results.

## Conclusion

In adults, after elective rheumatic heart valve surgery, the highest VIS in the immediate postoperative period is a good predictive value for EEx, with a threshold of 16.5.

### Electronic supplementary material

Below is the link to the electronic supplementary material.

Supplementary Material 1

Supplementary Material 2

Supplementary Material 3

## Data Availability

The data used and/or analyzed during the current study are also available from the corresponding author upon reasonable request.

## Declarations

**Ethics approval and consent to participate**.

The present study was designed as a single-center, retrospective, cohort study conducted for the period between January 2017 and December 2021. The study procedure was approved by the Medical Ethics Committee of the Affiliated Hospital of North Sichuan Medical College. The study is registered with the Chinese Registry of Clinical Trials (http://www.chictr.org.cn) (ChiCTR2300071659; 22/05/2023) and conducted in accordance with the Declaration of Helsinki (2013). The requirement for obtaining written informed consent from each participant was exempted from the Medical Ethics Committee of the Affiliated Hospital of North Sichuan Medical College due to the retrospective nature of the study that ensured no exposure risks to the patients due to any additional intervention.
