# Supplementary figures and images for: Availability and threshold of the vasoactive-inotropic score for predicting early extubation in adults after rheumatic heart valve surgery: a single-center retrospective cohort study
Source: BMC Anesthesiol. 2024 Mar 18;24:102. doi: 10.1186/s12871-024-02489-7 (PMC10946098; doi:10.1186/s12871-024-02489-7)

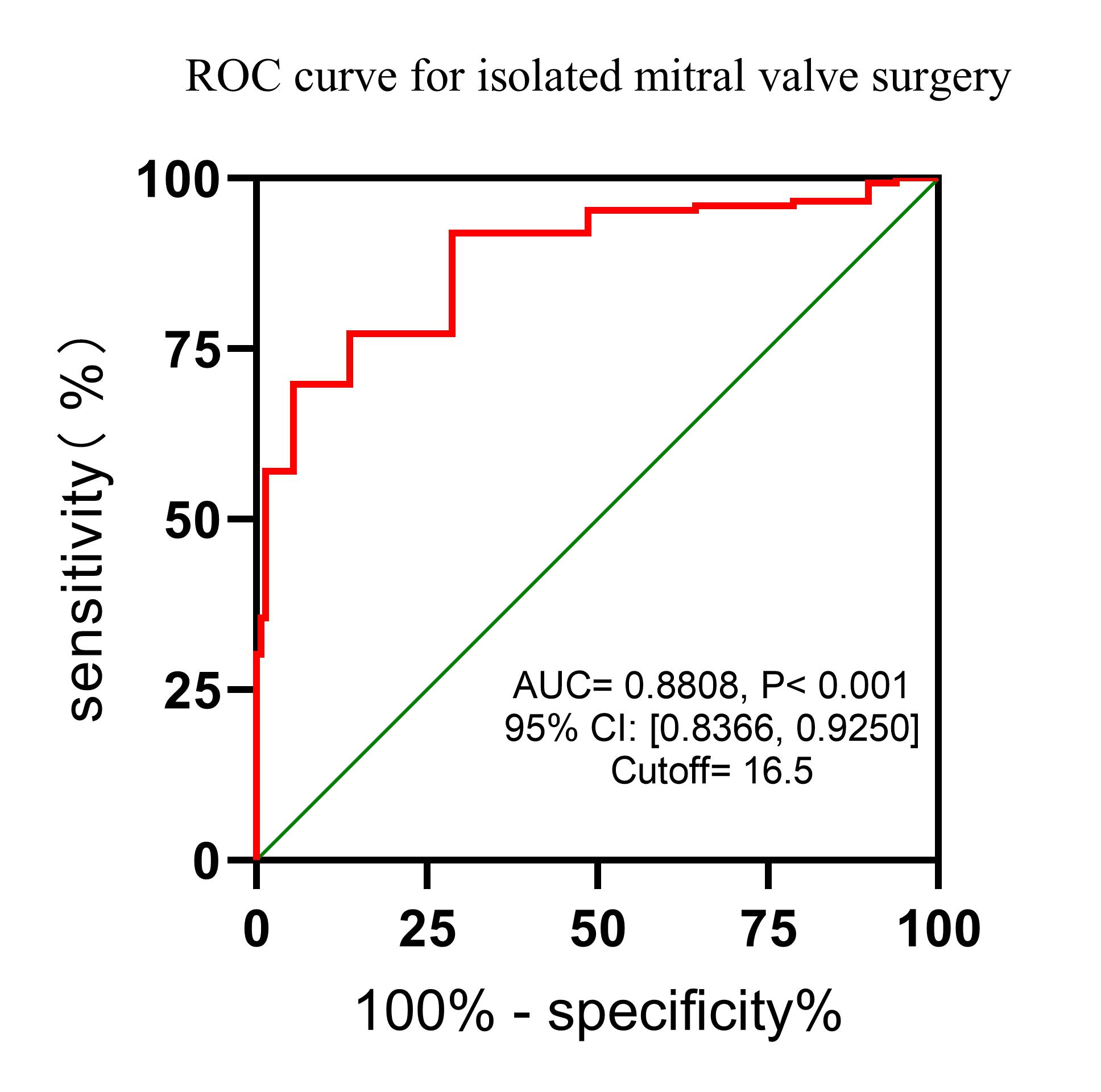

Supplement: Supplementary file 1 — Supplementary Material 1 [file 12871_2024_2489_MOESM1_ESM.jpg]

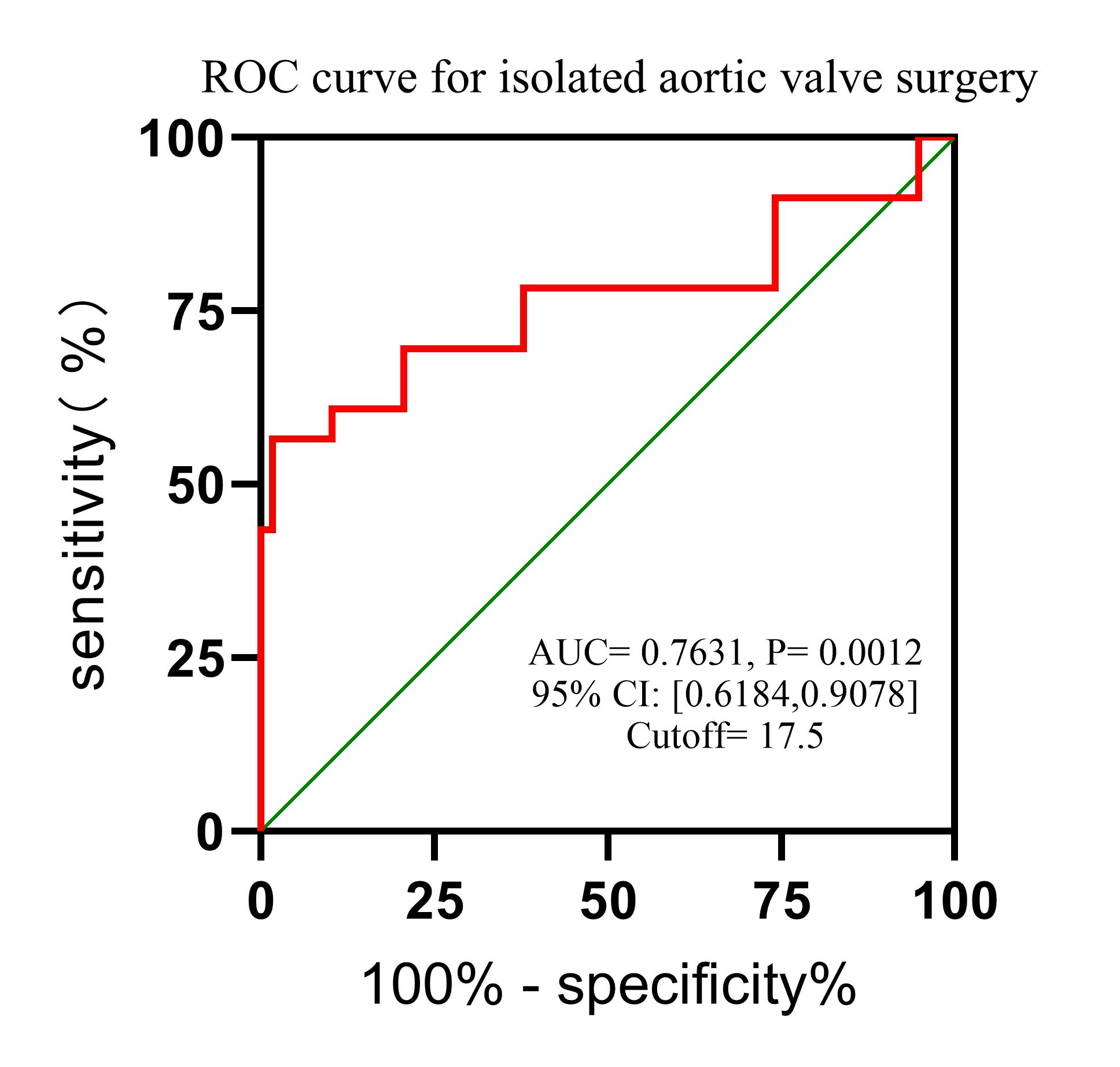

Supplement: Supplementary file 2 — Supplementary Material 2 [file 12871_2024_2489_MOESM2_ESM.jpg]

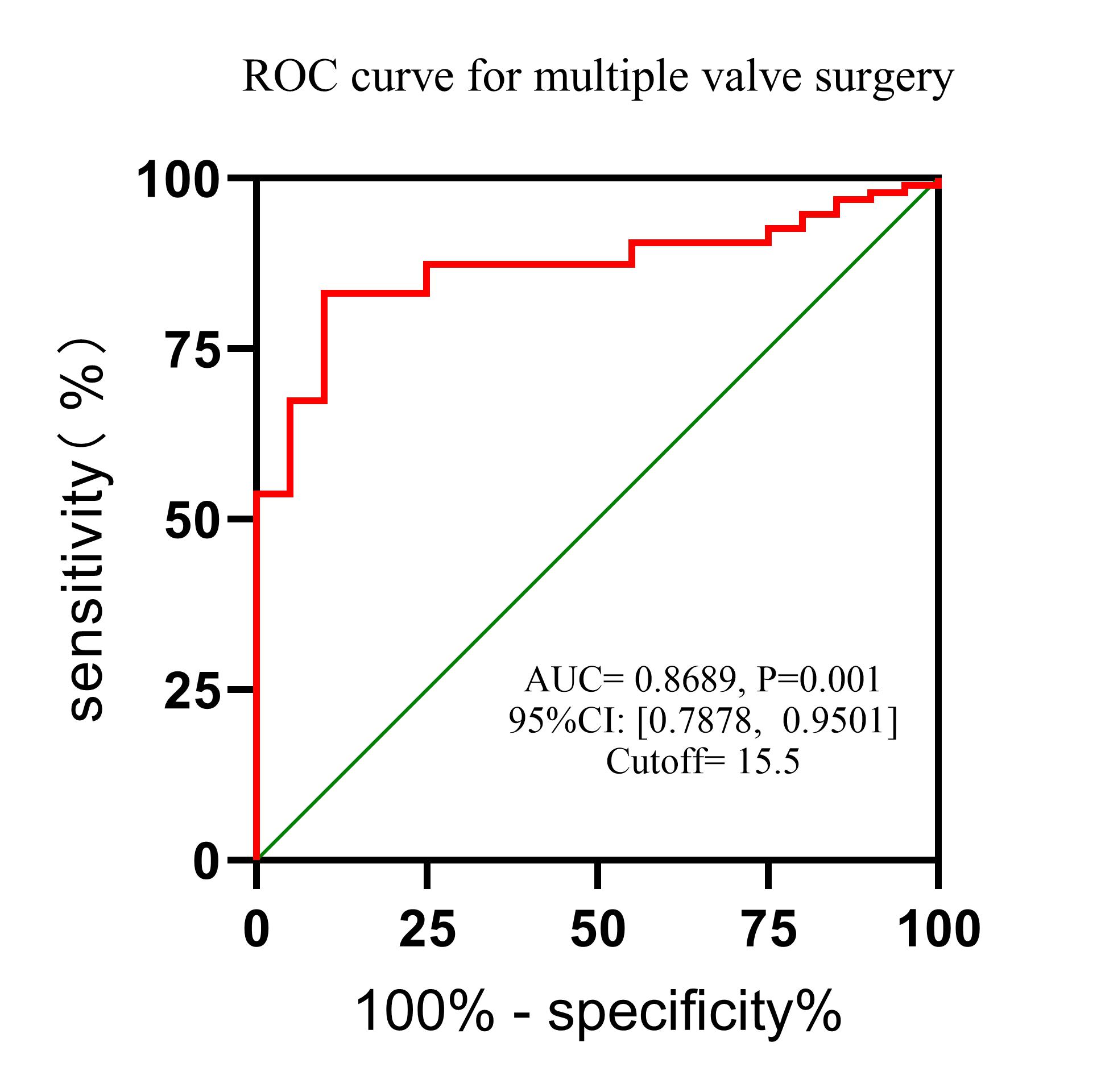

Supplement: Supplementary file 3 — Supplementary Material 3 [file 12871_2024_2489_MOESM3_ESM.jpg]
